# Antimicrobial Activity of Citrate-Coated Cerium Oxide Nanoparticles

**DOI:** 10.3390/nano14040354

**Published:** 2024-02-13

**Authors:** Ekaterina Vladimirovna Silina, Olga Sergeevna Ivanova, Natalia Evgenevna Manturova, Olga Anatolyevna Medvedeva, Alina Vladimirovna Shevchenko, Ekaterina Sergeevna Vorsina, Raghu Ram Achar, Vladimir Anatolevich Parfenov, Victor Aleksandrovich Stupin

**Affiliations:** 1 Department of Pathological Physiology, Sklifosovsky Institute of Clinical Medicine, I.M. Sechenov First Moscow State Medical University (Sechenov University), 119991 Moscow, Russia; parfenov_v_a@staff.sechenov.ru; 2 Frumkin Institute of Physical Chemistry and Electrochemistry, Russian Academy of Science, Leninskiy Pr., 31, Bldg. 4, 119071 Moscow, Russia; runetta05@mail.ru; 3 Department of Plastic and Reconstructive Surgery, Cosmetology and Cell Technologies, Pirogov Russian National Research Medical University, 117997 Moscow, Russia; manturovanatali@yandex.ru; 4 Department of Microbiology, Virology, Immunology, Kursk State Medical University, Karl Marx St, 3, 305041 Kursk, Russia; medvedevaoa@kursksmu.net (O.A.M.); shevchenkoav2@kursksmu.net (A.V.S.); vorsinaes@kursksmu.net (E.S.V.); 5 Division of Biochemistry, School of Life Sciences, Mysuru, JSS Academy of Higher Education and Research, Mysuru 570015, Karnataka, India; rracharya@jssuni.edu.in; 6 Department of Hospital Surgery No.1, Pirogov Russian National Research Medical University, 117997 Moscow, Russia; stvictor@bk.ru

**Keywords:** nanoparticles, cerium oxide, antimicrobial activity, nanoceria, nanocerium, gas chromatography, mass spectrometry, bacteriostatic effect, chemiluminescence, redox, peroxidase activity

## Abstract

The purpose of this study was to investigate the antimicrobial activity of citrate-stabilized sols of cerium oxide nanoparticles at different concentrations via different microbiological methods and to compare the effect with the peroxidase activity of nanoceria for the subsequent development of a regeneration-stimulating medical and/or veterinary wound-healing product providing new types of antimicrobial action. The object of this study was cerium oxide nanoparticles synthesized from aqueous solutions of cerium (III) nitrate hexahydrate and citric acid (the size of the nanoparticles was 3–5 nm, and their aggregates were 60–130 nm). Nanoceria oxide sols with a wide range of concentrations (10^−1^–10^−6^ M) as well as powder (the dry substance) were used. Both bacterial and fungal strains (*Bacillus subtilis*, *Bacillus cereus*, *Staphylococcus aureus*, *Pseudomonas aeruginosa*, *Escherichia coli*, *Proteus vulgaris*, *Candida albicans*, *Aspergillus brasielensis*) were used for the microbiological studies. The antimicrobial activity of nanoceria was investigated across a wide range of concentrations using three methods sequentially; the antimicrobial activity was studied by examining diffusion into agar, the serial dilution method was used to detect the minimum inhibitory and bactericidal concentrations, and, finally, gas chromatography with mass-selective detection was performed to study the inhibition of *E. coli’s* growth. To study the redox activity of different concentrations of nanocerium, we studied the intensity of chemiluminescence in the oxidation reaction of luminol in the presence of hydrogen peroxide. As a result of this study’s use of the agar diffusion and serial dilution methods followed by sowing, no significant evidence of antimicrobial activity was found. At the same time, in the current study of antimicrobial activity against *E. coli* strains using gas chromatography with mass spectrometry, the ability of nanoceria to significantly inhibit the growth and reproduction of microorganisms after 24 h and, in particular, after 48 h of incubation at a wide range of concentrations, 10^−2^–10^−5^ M (48–95% reduction in the number of microbes with a significant dose-dependent effect) was determined as the optimum concentration. A reliable redox activity of nanoceria coated with citrate was established, increasing in proportion to the concentration, confirming the oxidative mechanism of the action of nanoceria. Thus, nanoceria have a dose-dependent bacteriostatic effect, which is most pronounced at concentrations of 10^−2^–10^−3^ M. Unlike the effects of classical antiseptics, the effect was manifested from 2 days and increased during the observation. To study the antimicrobial activity of nanomaterials, it is advisable not to use classical qualitative and semi-quantitative methods; rather, the employment of more accurate quantitative methods is advised, in particular, gas chromatography–mass spectrometry, during several days of incubation.

## 1. Introduction

In recent decades, the development of technologies for producing nanoparticles using chemical or physical methods has caused a sharp surge in interest in these new materials from the scientific community. This is explained by the fact that nanoparticles and nanomaterials exhibit specific physical and chemical properties that are atypical for all materials [1,2,3,4]. This has even led to the proposal of separate issues related to the explanation of the action of nanoparticles into a separate subsection of nanoscience [5,6,7]. At the same time, concerns are being expressed that the widespread use of nanoparticles in various areas of human activity may have a detrimental effect on human health [8,9,10]. Therefore, in particular, biologists, doctors, chemists, and physiologists are interested in the study of nanoparticles. According to the PubMed electronic database, the number of articles devoted to the production and potential uses of cerium nanoparticles in medicine and veterinary science has increased over the past 10 years, from 156 in 2012 to 407 in 2022. Researchers have associated the most significant prospects for the use of nanoparticles with the noted increase in the regenerative activity of tissues during traumatic damage (including infected wounds of various etiologies), the restoration of functioning tissue after severe somatic diseases accompanied by the partial necrosis of organs [11,12,13,14,15], and antioxidant [16,17,18,19,20] and antitumor effects [21,22,23,24] with controlled, targeted drug delivery [25,26]. It is especially worth noting the close attention that has been paid in the modern medical and biological literature to the still difficult-to-predict antibacterial effects that accompany exposure to metal nanoparticles and metal salts with Gram-positive and Gram-negative bacteria [20,27,28,29,30,31,32].

In recent times, medical practice has been characterized by an increase in the number of patients with infected acute and chronic wounds or other skin lesions, which places an additional burden on the medical budgets of countries [33,34,35]. The reasons for this are domestic and military conflicts, as well as an increase in the number of patients with vascular and endocrine diseases [36,37]. The interest in the creation of new antimicrobial drugs is fueled by the increasingly widely discussed issues of the rapidly developing resistance of microorganisms to modern antibiotics, including the formation of bacterial films [38,39,40,41,42]; accordingly, this has led to a significant increase in hospital mortality [43,44].

There are studies in the literature that claim that nanoparticles of heavy metals or metals with variable valence have a pronounced antimicrobial effect [30,45,46,47,48,49,50]. At the same time, there are also works in which the authors did not obtain such an effect [51,52,53,54,55]. This contradiction is also noted in systematic reviews [51]; therefore, this allows us to state the absence of unambiguous conclusions about the presence of antimicrobial properties of nanoparticles whilst taking into account publication bias (data with identified effects are published more often, and in the absence of positive conclusions, works are not published; this can lead to fundamental erroneous system conclusions). It is difficult to unambiguously identify the reason for such fundamental discrepancies. It is highly probable that this is due to the methods of obtaining the nanoparticles and therefore to the resulting nanoparticles differing in physical and chemical properties, size, the structure of the particles and their chemical composition, environmental conditions, and surroundings [56,57,58,59,60]. This creates additional difficulties in the comparative analysis of the results of studies conducted by different authors, even if they have ostensibly used the same chemical substance.

As part of this work on the creation of dressings for the treatment of acute and chronic infected wounds, we chose to study cerium dioxide nanoparticles, as we found a large number of publications noting its antimicrobial [45,46,51,61,62] and regenerative properties [15,57,63,64,65,66,67].

In our previous works, we carried out a simultaneous study of the physicochemical characteristics and biomedical effects of nanocrystalline cerium oxide obtained by different methods (1—precipitation from aqueous solutions of cerium (III) nitrate hexahydrate and citric acid; 2—hydrolysis of ammonium hexanitratocerate (IV) under thermal conditions of autoclaving) with an assessment of the effect of different concentrations of nanocerium sols on the metabolic and proliferative activity of different layers of tissues involved in wound healing (on cell cultures of human fibroblasts, mesenchymal stem cells, and human keratinocytes). It has been proven that the method of synthesis of nanocrystalline cerium oxide and its concentration fundamentally change the biological activity of those cells providing wound regeneration. In addition, the citrate synthesis of nanocerium has shown the best biological activity in terms of the stimulation, metabolism, and proliferation of cell cultures involved in wound regeneration [57].

The purpose of this study is to investigate the antimicrobial activity of citrate-stabilized sols of cerium dioxide nanoparticles at different concentrations using various microbiological methods and comparing the effect with peroxidase activity for the subsequent creation of a regeneration-stimulating medical and/or veterinary product for wound healing providing new types of antimicrobial action.

## 2. Materials and Methods

The object of this study was citrate-coated cerium oxide nanoparticles (nanoceria), which we described in detail earlier [57,68].

Nanoceria sols were used in a wide range of concentrations (10^−6^, 10^−5^, 10^−4^, 10^−3^ and 10^−2^ M) as well as in powder form (dry matter).

### 2.1. Synthesis of Citrate-Coated Cerium Oxide Nanoparticles and Physicochemical Characterization

The synthesis of cerium dioxide sols stabilized by citrate ions was carried out from a highly alkaline medium (pH = 12) in the presence of citric acid as a stabilizer [57,68,69].

The main steps of the nanoceria synthesis were as follows:

(1) A total of 1.086 g of cerium(III) nitrate hydrate (Ce(NO_3_)_3_*H_2_O (>99.99%, LANHIT, Moscow, Russia) and 0.48 g of citric acid monohydrate (Sigma-Aldrich, C7129-100G, Batch, St. Louis, MO, USA) were mixed with 50 mL of distilled water.

(2) The mixture of cerium dioxide and citric acid in distilled water was added to 100 mL of ammonia solution (3 M) and stirred for 24 h at room temperature.

(3) After 24 h, 0.5 mL of the resulting solution was taken and diluted with distilled water at a ratio of 1:4 to 1:8 for spectrophotometry to determine the quality of the Ce^+3^-to-Ce^+4^ transition.

(4) An excess (350 mL) of isopropyl alcohol (≥99.5%, Chimmed, Moscow, Russia) was added to the resulting solution (in a measuring cup) to precipitate the cerium dioxide.

(5) Centrifugation was then carried out for 4–5 min at 19,000 rpm at room temperature. Excess isopropyl alcohol was drained off.

(6) The precipitate from the tubes was separated in an ultrasonic bath, transferred to a graduated cylinder, and the volume was brought to 50 mL with distilled water. A sample of 3 mL was taken for thermogravimetry.

(7) The concentration of the resulting sol was determined by the thermogravimetric method (3 mL of solution was added and heated for 3 h to 900 °C, the temperature plateau of 900 °C was maintained for 2 h, and then there was gradual cooling at a heating/cooling rate of 3–5 degrees/min).

(8) The remaining volume of the sol was transferred into a measuring beaker and placed on a magnetic stirrer at 60 °C for 40 min to evaporate the isopropyl alcohol (until the odor disappeared).

(9) The resulting sol was then diluted with distilled water to obtain the desired concentrations based on thermogravimetry data.

The resulting nanoceria were characterized by the following physicochemical methods.

To record the ultraviolet–visible absorption spectra, an SF-2000 spectrophotometer (OKB Spektr, Saint Petersburg, Russia), operating according to a single-beam scheme, was used. The recording was carried out in the wavelength range from 190 to 800 nm with a step of 0.1 nm, and the width of the optical slit was 0.2 nm. A recording in the range from 190 to 394.9 nm was carried out using a deuterium lamp, a recording in the range from 395 to 800 nm was carried out using a halogen lamp. The exposure time was 50 ms.

The particle size distribution and ζ-potential measurements of the cerium dioxide sols was analyzed with dynamic light scattering (DLS) using a Zetasizer Nano ZS laser analyzer with a 633 nm laser (Malvern Instruments Limited, Malvern, Worcestershire, UK). The sample preparation was carried out using Milli-Ω Water (18.2 MΩ/cm), and a temperature equilibrium was ensured between the sample cell and the cuvette holder. The correlation function for each of the samples was gained by averaging 10 curves, each being acquired for 20 s. Then, the data were filtered by adjusting the permissible deviation of the scattering intensity from the average value (no more than 10%), taking into account the shift of the baseline.

The powder X-ray diffraction analysis of the samples was carried out on a Rigaku D/MAX 2500 diffractometer (CuKα-radiation) at a goniometer rotation speed of 1–2 °2θ/min (Rigaku Corporation, Tokyo, Japan). The identification of diffraction maxima was carried out using the International Centre for Diffraction Data (Joint Committee on Powder Diffraction Standards (JCPDS) data bank, PA USA). The particle size was estimated using the Scherrer approach as it is quite simple and it can be used for X-ray diffraction patterns with small number of X-ray maxima, which is the case for cubic ceria.

The transmission electron microscopy (TEM) of the synthesized samples was carried out on a JEM 2100 JEOL electron microscope (JEOL Ltd., Tokyo, Japan). The accelerating voltage was 200 kV.

### 2.2. Study of the Intensity of Chemiluminescence (Peroxidase Activity) of Cerium Dioxide Sols at Different Concentrations

The enzyme-like activity (peroxidase/catalase) of the cerium dioxide sols was studied in a model reaction of luminol oxidation in the presence of hydrogen peroxide in a phosphate buffer solution (KH_2_PO_4_ (Sigma #1.04873), c = 100 mM, pH 7.4). At this pH value, an acceptable quantum yield of luminol chemiluminescence could be obtained in the reaction mixture containing hydrogen peroxide at a concentration of 10 mM. A solution of luminol (5-amino1,2,3,4-tetrahydro-1,4-phthalazinedione, 3-aminophthalic acid hydrazide, Sigma #123072, c = 1 mM) was prepared by dissolving a sample of luminol (0.0885 g) in a phosphate buffer solution (500 mL). A working solution of hydrogen peroxide with a concentration of 1 M was prepared by diluting a 30% H_2_O_2_ solution (special-purity-grade, Khimmed, Russia) with distilled water. The registration of chemiluminescence (CL) was carried out in plastic cuvettes with a volume of 2 mL on a 12-channel chemiluminometer Lum-1200 (DISoft, Moscow, Russia) using the PowerGraph software (version 3.3). The analytical signal was recorded under temperature control (37 °C) directly in the cuvette compartment of the chemiluminometer. Aliquots of luminol (c = 50 µM) and H_2_O_2_ (c = 200 µM) were added to a plastic cuvette containing a phosphate buffer solution (100 mM). The background glow was recorded for 60 s; then, an aliquot of the analyzed CeO_2_ sol was added. Various concentrations of CeO_2_ in the reaction mixture (10^−3^–10^−5^ M) were studied. The total volume of the reaction mixture was 1 mL. The higher the chemiluminescence intensity, the more reactive the oxygen species were in the system. The integral intensity (light sum) of chemiluminescence, which depended less on the measurement conditions than the absolute intensity, was also used as an analytical signal. The mathematical modeling of the kinetics of the chemiluminescence of luminol in the presence of hydrogen peroxide and cerium dioxide was carried out using the Kinetic Analyzer software (version 3.1).

### 2.3. Microbiological Methods

Each sample of citrate-stabilized nanoceria sol at various concentrations was examined sequentially using three methods employed in the assessment of antimicrobial activity: (1) the agar diffusion method; (2) the method of serial dilutions in meat peptone broth in order to identify the minimum inhibitory concentration; and (3) the method of gas chromatography with mass selection.

#### 2.3.1. Determination of Antimicrobial Activity by Agar Diffusion Method

Since we were in the process of developing a medicine, we initially carried out antimicrobial studies using the classical agar diffusion method for clinical practice. This is the method recommended by the Russian State Pharmacopoeia of our country for studying the antimicrobial activity of antibiotics, antiseptics, medicinal plant materials, and newly synthesized compounds. The method was performed on a solid nutrient medium; zones of growth inhibition of test microorganisms used to determine the antimicrobial effect of medicinal substances were analyzed.

The study groups were citrate-stabilized nanoceria in five concentrations (10^−2^, 10^−3^, 10^−4^, 10^−5^, and 10^−6^ M).

For the control and comparison groups, water for injection (negative control) was used as a control. For comparison, the positive controls were used. The antibiotic ceftriaxone, in a solution of 0.25 g/mL (Promomed, Russia, Moscow), and an ointment for external use, which was levomekol containing dioxomethyl-tetrahydropyrimidine 40 mg/g + chloramphenicol 7.5 mg/g (Nizhpharm, Nizhny Novgorod, Russia), were used.

All substances of all groups were added to the agar in the same volume (0.1 mL).

Each sample was examined at least 5 times using test strains of aerobic, facultative aerobic bacteria and fungi from the collection of the Research Institute for Standardization and Control of Medical Biological Preparations named after A.A. Tarasevich (Russia, Moscow). The test strains of the microorganisms that were used for this study were as follows: *Bacillus subtilis* ATCC 6633, *Bacillus cereus* ATCC 10702, *Staphylococcus aureus* ATCC 6538, *Pseudomonas aeruginosa* ATCC 9027, *Escherichia coli* ATCC 8739, *Proteus vulgaris* ATCC 4636, *Candida albicans* AT CC 10231, and *Aspergillus brasielensis* ATCC 16404.

The cultures of the test strains of microorganisms were grown on a solid medium (meat peptone agar) at a temperature of 37 °C ± 2 °C for 18–20 h. The microbial load was prepared by diluting the test culture microorganisms with a sterile isotonic sodium chloride solution using a turbidity standard (billion suspension).

For the experiments to determine antimicrobial activity, a microbial load was chosen—500,000 microorganisms per 1 mL.

A total of 0.2 mL of a 1,000,000,000 suspension of microorganisms was added to 400 mL of meat peptone agar heated to 49 °C ± 1 °C; then, 15 mL was poured into sterile Petri dishes, so the final microbial load was 500,000 microorganisms in 1 mL.

Petri dishes with the frozen inoculated medium were thermostatted to remove the condensate, after which wells with a diameter of 7 mm were cut out under sterile conditions. A total of 0.1 mL of the test sample was added to each well. The dishes were maintained at room temperature for 1 h; then, all the Petri dishes were incubated at 36 °C ± 1 °C for 17 ± 1 h. After the specified period of incubation in the thermostat, the zones of growth inhibition of the test microbes were measured, expressing the results in mm. Considering the socket diameter of 7 mm and a 1 mm error zone around it, a zone of less than 10 mm was considered as a zone with no inhibition of microorganism growth. Thus, this technique determined the presence or absence of zones of growth inhibition; then, if present, the quantitative value of the size of the zone of growth inhibition of the microorganisms was assessed.

#### 2.3.2. The Method of Serial Dilutions for Determination of the Minimum Inhibitory Concentration and Minimum Bactericidal Concentration of Nanoceria

Nanoceria were studied to determine the minimum inhibitory concentration (MIC) and minimum bactericidal concentration (MBC) using the following strains of microorganisms: Bacillus subtilis ATCC 6633, *Staphylococcus aureus* ATCC 6538, *Escherichia coli* ATCC 8739, and *Candida albicans* ATCC 10231. Cultures of the microorganisms were grown on a solid medium at a temperature of 37° ± 2 °C for 18–20 h. The initial microbial load was 500,000 microorganisms per 1 mL.

For this study, 11 test tubes were provided with 1 mL of meat peptone broth (MPB) in each tube. Then, 1 mL of nanoceria starting solution at a concentration of 0.1 g/mL was added to test tube No. 1 (maximum nanoceria concentration: 0.05 g/mL) and mixed with MPB; next, 1 mL of the solution was transferred from test tube No. 1 to test tube No. 2, mixed, and then 1 mL of the solution was transferred from test tube No. 2 to test tube No. 3, and mixed. This procedure was repeated exactly for each test tube until reaching test tube No. 10, from which 1 mL of the solution was poured. Test tube No. 11 was a control tube, into which the test substance was not added. Thus, the volume of solution in each test tube was 1 mL. The next step was to add a microbial suspension of 0.05 mL to each test tube, including the control one.

Since this method requires the presence of a maximum concentration of the test substance, 1 g of dry nanocerium oxide was used for this study. To prepare the cerium oxide concentrations, 1 g of nanocerium powder was weighed out, placed in a 10 mL flask, 5 mL of distilled water was added, and the flask was shaken on a laboratory vortex for 10 min; then, the volume was adjusted to the mark with distilled water, and the mixture was shaken again on a laboratory vortex for a further 10 min. A concentration of 1 g/10 mL was obtained (the suspension was thoroughly shaken before performing the MIC study). As a result, in test tube No. 1, the concentration of nanoceria was at a maximum and amounted to 0.05 g/mL; in test tube No. 2, it was 2 times less, at 0.025 g/mL; in No. 3, it was 0.0125 g/mL; in No. 4, it was 0.0063 g/mL; in No. 5, it was 0.0031 g/mL; in No. 6, it was 0.0016 g/mL; in No. 7, it was 0.0008 g/mL; in No. 8, it was 0.0004 g/mL; in No. 9, it was 0.0002 g/mL; and in No. 10, it was 0.0001 g/mL.

The tubes were incubated in a thermostat at 37 °C for 24 h, after which the result was assessed.

According to the method for determining the MIC and MBC, the next step was performed after 24 h. Sterile Petri dishes containing 15 mL of meat peptone agar (MPA) without microorganisms were divided into sectors (the bottom of the dish) according to the number of test tubes. The contents of the tubes (1–11) were extracted using a bacterial loop under aseptic conditions (laminar flow hood) and inoculated onto the corresponding sector of a Petri dish. The crops were incubated in a thermostat at 37 °C for 24 h, after which the final results (the qualitative indicator) were assessed.

#### 2.3.3. Methodology for Determining the Effect of Nanoceria on the Reproduction of Microorganisms by Mass Spectrometry of Microbial Markers Using a Gas Chromatograph with a Mass-Selective Detector

The next stage of this work was the analysis of different concentrations of nanoceria using a gas chromatograph with a mass-selective detector “Maestro” (Interlab, Russia, Moscow) assessing the growth inhibition of *Escherichia coli* ATCC 8739 (*E. coli*). This method is based on a highly accurate determination of the presence of molecular characteristics of markers specific to specific microorganisms (higher fatty acids, aldehydes, alcohols, and sterols in the test sample); indeed, the fatty acid status of the microorganisms was used, which is specific and genetically determined.

The initial concentration was 500,000 microorganisms in 1 mL of culture medium.

The studied concentrations of the nanoceria sols were 10^−2^, 10^−3^, 10^−4^, and 10^−5^ M.

The control and comparison groups were as follows: (1) culture medium (CM): here, the test tubes contained only MPB in a volume of 5 mL; (2) culture medium: this comprised 4.5 mL + *E. coli* suspension of 0.5 mL (CM + *E. coli*); (3) culture medium: this comprised 4.0 mL + *E. coli* suspension of 0.5 mL + 0.5 mL of water (CM + *E. coli* + H_2_O); (4) the reference for comparison of the antimicrobial activity was ceftriaxone (0.25 g/mL, the same as in Section 2.3.1.), which was added in a volume of 0.5 mL into the CM (4.0 mL) + *E. coli* suspension of 0.5 mL.

The study groups were as follows: the test tubes Nos. 5, 6, 7, and 8 contained nanoceria sols of different concentrations (10^−2^ M, 10^−3^ M, 10^−4^ M, and 10^−5^ M, respectively) in a volume of 0.5 mL, which were added to the concentration of CM of 4.0 mL + *E. coli* 0.5 mL (CM + *E. coli* + CeO).

The research technique using a gas chromatograph was performed as follows. A sample with a volume of 40 μL was extracted from the test tubes using a dispenser, placed in a vial, and acid methanolysis was carried out at a temperature of 80 °C for 45 min. At the end of the acid methanolysis, the vial was cooled; then, 400 μL of hexane was added to the test sample, and the mixture was shaken on a vortex for 1 min (extraction). Next, 5 min after shaking, the separation of the methanol/hexane layers was observed, and 200 μL of the upper hexane layer was extracted with a dispenser and placed in clean vial. The vial with the hexane phase was placed in a thermostat (T = 80 °C) for 7 min to evaporate the hexane. After the evaporation of the hexane, the vial was cooled, 20 μL of N,O-bis(trimethylsilyl)-trifluoroacetamide (BSTFA) was added to the dry residue, and the vial was closed with a lid and heated in a thermostat (T = 80 °C) for 5 min. The vial was cooled; then, 60 μL of hexane was added to the reaction mixture, the total volume of the solution—80 μL—was transferred into a conical insert, the insert was placed in the same vial, and the vial was closed with a lid. The resulting sample was subjected to gas chromatographic–mass spectrometric analysis.

Chromatographic separation and scanning were then carried out. The separation was carried out on a 5%-phenyl-95%-methylsiloxane column of 25 m × 0.25 mm with a phase thickness of 0.25 μm in a flow of helium carrier gas. The heating rate of the column thermostat was 7 °C/min in the range of 125–320 °C. The evaporator temperature was 280 °C in the split-flow mode. The interface temperature was 280 °C, and the quadrupole temperature was 150 °C. Ionization was carried out via electron impact with an ionization energy of 70 eV. The detection took place in the selective ion mode with periodic scanning of up to thirty ions in five time intervals. The time intervals and registered ions were initially specified in the method; for an in-depth study of the principles of the formation of intervals and groups of ions, please refer to the description of the medical technology and original patents. The prepared and labeled samples were installed in the autosampler carousel.

A series of tubes were placed in a thermostat (T = 37 °C) for 24 h. Each sample was examined at least 5 times.

The final calculation of the number of microorganisms was expressed as the number ×10^5^ microbial cells per 1 g of the studied material.

### 2.4. Statistical Analysis

For the creation of the graphs and to analyze the data of the nanoceria physicochemical characterization, OriginPro 2018 from OriginLab software SR1 (Northampton, MA, USA) was used.

The statistical processing and creation of the graphs of the microbiological quantitative results of this study were carried out using the statistical program software SPSS 25.0 (IBM Company, New York, NY, USA). The normality of the distributions of the GC-MS indicators (number of microorganisms, ×10^5^ cells) was assessed using the Kolmogorov–Smirnov and Shapiro–Wilk tests. All quantitative variables had a normal distribution. Descriptive statistics of continuous quantitative indicators that were subject to normal distribution are presented as the mean, std. deviation, std. error, 95% confidence interval for the mean (95CI), minimum, and maximum. For the comparative analysis of the different test subgroups, one-way analysis of variance ANOVA was performed. Post hoc multiple comparisons were performed using Dunnett’s tests (for comparison with controls) as well as Bonferroni’s test. The number of repetitions (sample) was at least 5; taking this into account, all comparison results were rechecked using Mann–Whitney’s test. Given the multiple comparisons, differences were considered statistically significant at a *p*-value < 0.01.

## 3. Results

### 3.1. Physicochemical Characterization

**Figure 1:**
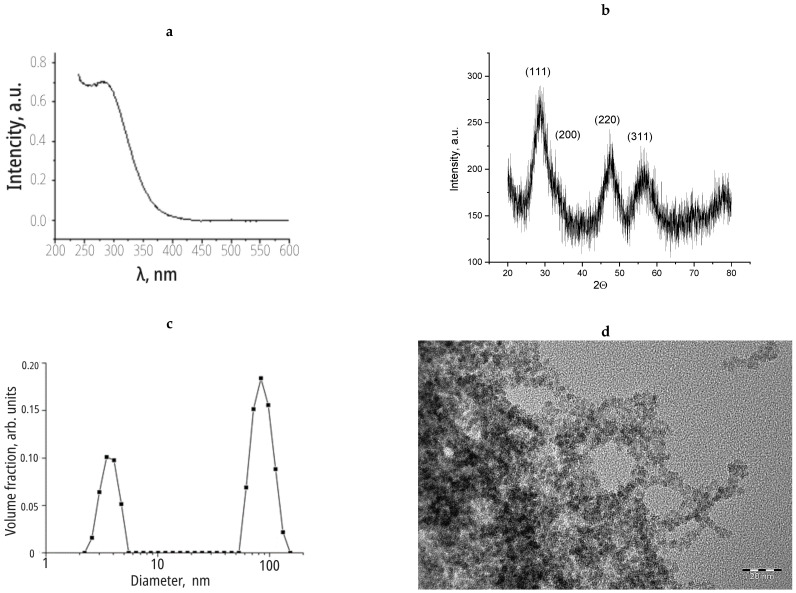
(**a**) The results of analysis of nanoceria sol via spectroscopy methods; (**b**) X-ray diffraction; (**c**) dynamic light scattering; (**d**) transmission electron microscopy.

The X-ray diffraction data allowed for estimating the particle size of the cerium dioxide using the Scherrer approach as 3–5 nm, which was in good agreement with the TEM data (Figure 1). According to the dynamic light scattering results, the colloidal solution of cerium dioxide included both individual particles with a size of 5 nm and aggregates of particles whose diameter ranged from 60 to 120 nm.

### 3.2. Results of Chemiluminescent Analysis of Cerium Dioxide Stabilized with Citrate Ions

**Figure 2:**
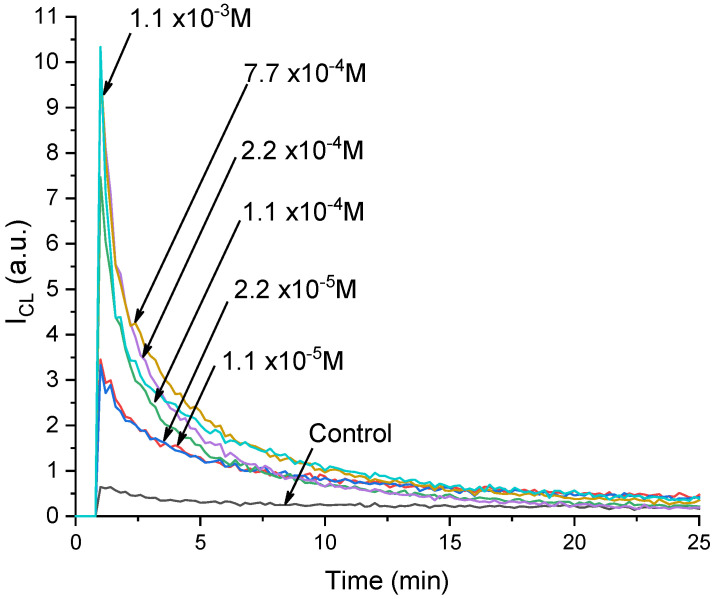
Kinetic curves of chemiluminescence of the product of luminol oxidation with hydrogen peroxide in a reaction mixture containing a phosphate buffer solution (pH 7.4) and cerium dioxide citrate sols. The concentrations of CeO_2_ in the reaction mixture are shown in the figure.

The addition of citrate-stabilized cerium dioxide sols at different concentrations led to a proportional increase in the chemiluminescence intensity (Figure 2). This means that the cerium dioxide exhibited a peroxidase-like activity and thereby catalyzed the oxidation reaction of luminol with hydrogen peroxide, which caused an increase in the luminescence.

**Figure 3:**
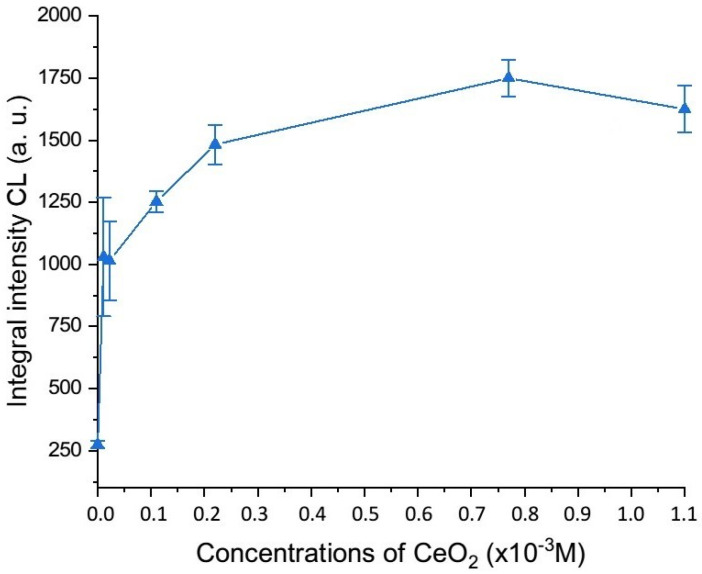
Dependence of the integral intensity of chemiluminescence of the oxidation products of luminol on the concentration of cerium dioxide in reaction mixtures containing a buffer solution (pH 7.4), luminol, hydrogen peroxide, and CeO_2_ sols coated with citrate ions.

Integral intensities were calculated from the chemiluminescence curves recorded for the samples of CeO_2_ sols stabilized with citrate ions. The results of the quantitative analysis of the enzyme-like activity of the cerium dioxide sols are shown in Figure 3.

It was established that the peroxidase-like activity of the sols of cerium dioxide nanoparticles coated with citrate naturally increased in proportion to the increase in the CeO_2_ concentration from 1.1 × 10^−5^ M to 2.2 × 10^−4^ M; then, the increase in the chemiluminescence intensity was less pronounced, with a maximum peak at a concentration of about 1 × 10^−3^ M.

### 3.3. Results of the Study of Antimicrobial Activity by the Agar Diffusion Method

**Figure 4:**
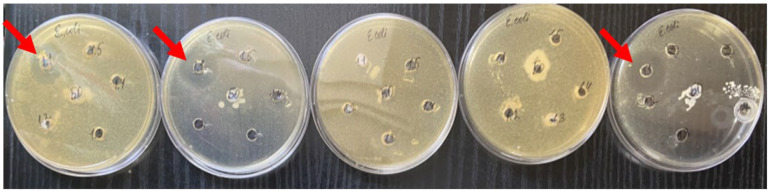
Zones of growth inhibition for *E. coli* in three samples of nanoceria with a maximum concentration of 10^−2^ M. The red arrows indicates obvious areas of growth retardation.

Following the extensive study of the antimicrobial activity of different concentrations of cerium dioxide citrate nanoparticles, the results of this study, following a recognized methodology, did not show an absolute antimicrobial effect, such as would be characteristic of pharmacopoeia standards for antibiotics and antiseptics (Table 1). However, some features were identified that confirmed the presence of antimicrobial activity of the samples. Thus, the inconsistent antimicrobial activity of the nanocerium sol at a concentration of 10^−2^ mol/L against *E. coli* was determined; in three out of the five Petri dishes (60%), a growth inhibition zone was detected from 16 mm to 25 mm, and on average, it was 19 mm (Figure 4).

### 3.4. Determination of the Minimum Inhibitory Concentration and Minimum Bactericidal Concentration of Cerium Oxide Coated with Citrate

**Figure 5:**
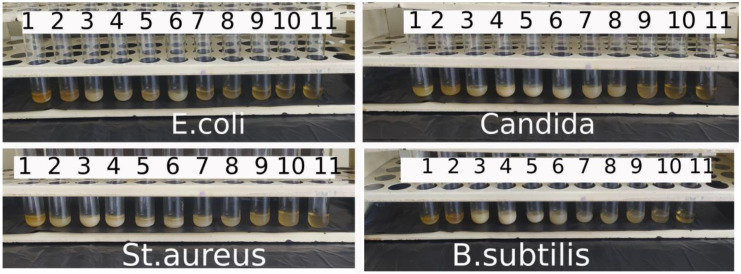
Results of the first phase of the experiment to determine the minimum inhibitory concentration of citrate-stabilized nanoceria against various microorganisms. The numbers indicate the numbers of the tubes, in test tube No. 1 the concentration of nanocerium is maximum (0.05 g/mL), in test tube No. 2—2 times less (0.025 g/mL) and so on, in test tube No. 10 the concentration of nanocerium is minimal (0.0001 g/mL); test tube No. 11—Control.

After 24 h of incubation in a thermostat of a series of test tubes with different concentrations of nanoceria, it was visualized that the solution was cloudy in all the test tubes containing microorganisms (medium turbidity). This may indicate the growth of microbial flora. However, it was found that the color of the contents of the tubes changed (Figure 5; Table 2).

Compared to the 11th control tube, samples 3–6 with *E. coli*, 3–6 with Candida, 3–7 with *B. subtilis.*, and 2–8 with *St. aureus* acquired a white color. That is, for all the microorganisms, a whitish tint appeared at concentrations of citrate-coated nanoceria in the dose range of 0.0016–0.0125 g/mL (1.5–12.5 mg/mL; ~0.009–0.073 mol/L considering the molar mass of cerium oxide(IV) of 172.1 g/mol; that is, 10^−1^–10^−2^ M). This may be due to a bacteriostatic effect, maximized at these concentrations, where a part of the microorganisms died, giving a protein precipitate from the microbial bodies, but the remaining part of the living microorganisms retained the ability to multiply. This was shown by seeding.

**Figure 6:**
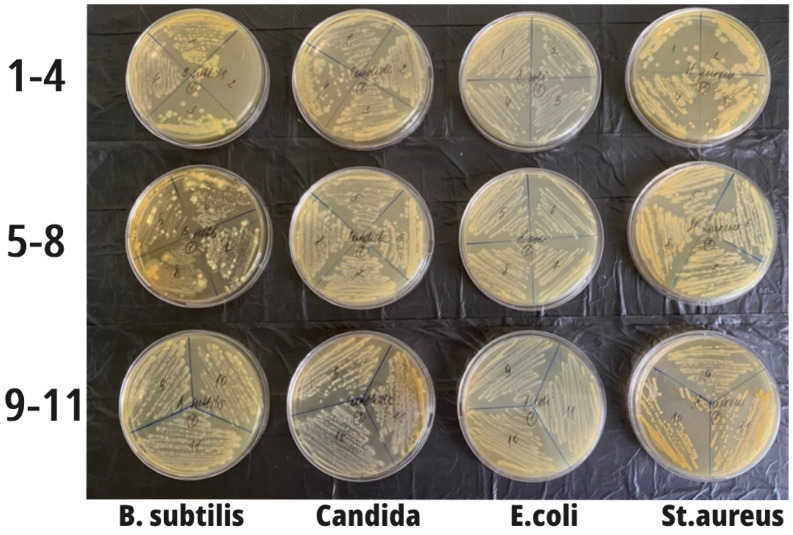
The growth of microorganisms in all Petri dishes. In the lower samples (the least concentrated and 11—the control), the growth intensity was maximum (on the left in the figure are samples with *B. subtilis*; in sector 2, there was almost complete inhibition of bacilli growth; in samples 3–7, the growth intensity was less pronounced compared to the control). The second row contained *Candida*, and growth inhibition was visualized in sectors 4–5. *E. coli* growth was reduced in sectors 2–3, and in *St. aureus*, growth was reduced in sectors 1–3.

According to the method employed, the contents of all eleven test tubes were then inoculated onto the corresponding sector of a Petri dish with sterile meat peptone agar. After 24 h of incubation of these crops in a thermostat at 37 °C, the growth of microorganisms was detected on all the Petri dishes (Figure 6). However, this growth was expressed differently; namely, in relation to *B. subtilis*, there was almost a complete delay in the growth of bacilli in sector 2. In samples 3–7, the growth intensity was less pronounced compared to the control. In the second row with Candida, growth inhibition was visualized in sectors 4–5. In relation to *E. coli*, a decrease in growth in sectors 2–3 was visualized, and in relation to *St. aureus*, a decrease in growth was seen in sectors 1–3.

Although the absolute antimicrobial effect has not been established (MIC and MBC have not yet been determined), at the same time, a tendency toward some inhibition of the growth of microorganisms was determined, mainly in test tubes 2, 3, and 4 (0.006–0.025 g/mL (0.04–0.14 M) citrate-stabilized cerium oxide nanoparticles).

### 3.5. Antimicrobial Activity of Sols of Citrate-Stabilized Cerium Oxide Nanoparticles in Different Concentrations according to Gas Chromatography with Mass Selection

**Figure 7:**
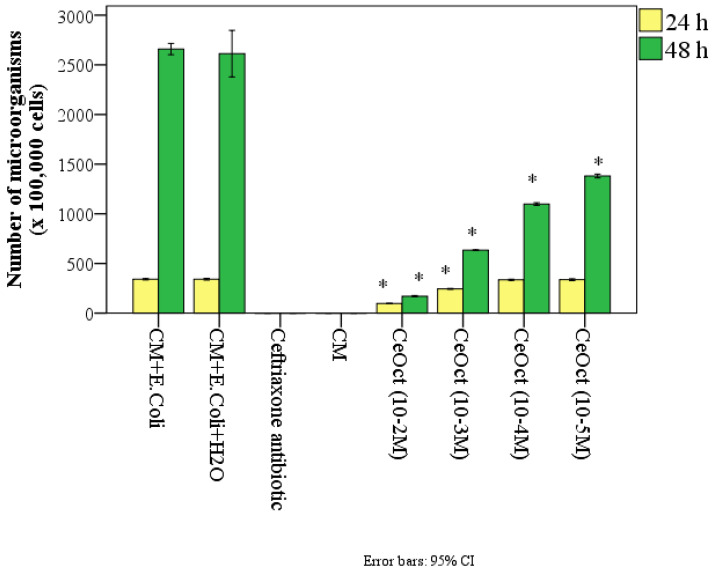
The number of microbial bodies of *E. coli* in different control groups and during co-cultivation with 10 vol.% sols of citrate-stabilized cerium oxide nanoparticles in different concentrations after 24 h and 48 h (*—significant difference from the control at *p* < 0.01; ANOVA and Dunnett’s and Bonferroni’s post hoc tests).

During this study, using gas chromatography with mass-selective detection, it was found that the nanoceria had a significant effect on the number of *E. coli*, showing a bacteriostatic effect (Figure 7).

Thus, after 24 h of the co-cultivation of microorganisms in a nutrient medium with the nanoceria sols, a statistically significant inhibition of *E. coli* growth was determined at high concentrations of nanoceria (10^−2^–10^−3^ M). Thus, in the control groups, where there was only a nutrient medium and *E. coli*, the average number of microbes per 1 g of test material was 341 ± 6.7 × 10^5^ cells. On average, it was 3.6 times more than in the samples with the addition of nanoceria at a concentration of 10^−2^ M and 1.4 times more at a concentration of 10^−3^ M (*p* < 0.01). Accordingly, the percentage of significant suppression of *E. coli* growth when co-cultured with nanoceria added to the test tube at a concentration of 10^−2^ M after 24 h was 72%, and at 10^−3^ M, it was 28% (*p* < 0.01). Other concentrations of citrate-coated nanoceria (10^−4^–10^−5^ M) after 24 h did not show a significant effect on the number of *E. coli.*

According to the gas chromatography results, on average, the number of *E. coli* increased significantly after 48 h by 7.8 times in the CM + *E. coli* samples and 7.7 times in the CM + *E. coli* + 10 vol% H_2_O samples relative to the 24th hour and, on average, by up to 2659 ± 50 and 2613 ± 223 × 10^5^ of the microorganisms, respectively.

After 48 h, a significant bacteriostatic effect of CeOct was revealed at all the concentrations that we studied, and a linear dose-dependent effect was established. In the control group, the number of microorganisms was significantly greater than in the nanoceria groups, which was, on average, greater by 15.6 times at a concentration of 10^−2^ M, 4.2 times at a concentration of 10^−3^ M, 2.4 times at a concentration of 10^−4^ M, and 1.9 times at a nanoceria concentration of 10^−5^ M (*p* < 0.001) (Table 3).

The use of the antibiotic ceftriaxone completely killed the microorganisms; no *E. coli* bacteria were found in any of the samples.

One-way analysis of variance (ANOVA) established the significance of multiple differences both after 24 h (F = 3326.5, *p* < 0.001) and after 48 h (F = 476.9, *p* < 0.001).

## 4. Discussion

Considering the growing global problem of antibiotic resistance, the antibacterial effects of developed nanomaterials are of the greatest interest. The long and widespread use of antibiotics has led to the emergence of antibiotic-resistant microorganisms and has created many problems for the medical community, including nosocomial infection [39,40,41,42,70]. Cerium oxide nanoparticles are of great potential interest as a new type of antimicrobial agent. However, the current work on the use of nanoceria as an antimicrobial agent for the treatment of wound and burn surfaces still poses a significant number of new questions.

The antimicrobial mechanism of action of nanoceria has not yet been fully elucidated; the mechanism remains debatable. The main theory is that of oxidative stress. Due to the massive production of reactive oxygen species in cells under the influence of CeO_2_ nanoparticles, there is an excess of active radicals that block the thiol groups of membrane proteins, causing their denaturation. As a result, the functionality of the microorganisms’ membranes is disrupted, which leads to their death [46,71].

Our results of the chemiluminescence analysis with the determination of the intensification of reactive oxygen species and the redox status of the nanoceria confirmed this theory. However, the first results of our work, obtained using the agar diffusion method, did not cause despair within the working interdisciplinary group of researchers, but they forced us to intensively discuss and assess the possible reasons for the antimicrobial effects that are proposed in other studies [45,46,47,48,49,50,51,62]. Since our scientific research group included chemists, biophysicists, microbiologists, biologists, pathophysiologists, pharmacologists, and surgeons, the search for the causes of this phenomenon was carried out across an extensive range of clinical and practical expertise. Before starting our microbiological studies, we were almost confident of a positive result, since we had studied the literature data demonstrating that nanoparticles of heavy metals or metals with variable valence have a pronounced antimicrobial effect [30,47,48,49,50]. However, our attempts to obtain a similar effect and then to compare the nanoparticles we synthesized with the nanoparticles used by other researchers who obtained a good antibacterial effect were in vain, since the articles we reviewed in most cases did not contain a detailed method for the synthesis of nanoceria. An exact description of the physicochemical properties of the nanoceria particles themselves was also not provided, which means that the very possibility of accurately comparing the results was called into question. Thus, our findings replicated those of a review regarding the conflicting antimicrobial results of nanoceria [51]. Despite the available literature data on the antibacterial effect of nanoceria, including against *E. coli*, an antibacterial effect of the nanoparticles was not always obtained. No antibacterial activity at all was observed in several experiments, including against *E. coli* [54,72,73,74,75].

This could be due to several factors. Many researchers have noted the dependence of the effects of nanoceria on the size of the particles themselves [76], perhaps due to the surface area, but possibly due to some other factors that increase the biocompatibility. Some researchers have argued that the green synthesis of nanoceria is not only environmentally friendly but also enhances the bactericidal effects against Gram-positive and Gram-negative bacteria [62,77,78]. We also wrote earlier that methods for the synthesis of nanoceria, even with a formal coincidence of particle sizes, provide different biological effects; in particular, this effect occurs when working with cell cultures [57].

The authors suggest that the classical seeding did not detect growth retardation (a lack of antimicrobial effect) due to the fact that nanoparticles of heavy metal cerium, not being a solution but a sol, simply do not diffuse into solid agar, or do so with a sufficiently long delay and under certain conditions. Thus, the agar diffusion method, when assessing the antimicrobial effect of nanoparticles, can be used only with a certain caution. The results of further studies revealed the bacteriostatic effect of the nanoceria. Moreover, the number of microbial bodies varied depending on the concentration of the nanoceria. A bacteriostatic effect does not mean complete death (bactericidal) of microorganisms. It means that despite a significant suppression of microorganism growth, live bacterial bodies still remained in the meat-peptone broth. These are the ones that gave positive cultures (growth of microorganisms) on the agar-agar. That is why we additionally carried out verification tests and confirmed this effect. Signs of microorganism multiplication were detected, but to a much lesser extent. Therefore, we can confidently assert that our synthesized nanoceria had a significant bacteriostatic antimicrobial effect (on the example of the *E. coli* strain).

The resulting bacteriostatic effect was pleasing, first of all, because it was persistent throughout the entire 48 h observation period. Moreover, the increase in this effect over time (which is not typical for current clinical antiseptics) allowed us to assume (and therefore justify our following protocols) that after 72 h and beyond, the antimicrobial effect may further significantly increase. Focusing only on the literature data and the protocols accepted in microbiology, we were not able to predict this precise effect. Although it was not possible to achieve absolute bactericidal activity, the number of microbial bodies increased by the end of 2 days. It is very likely that this effect is prolonged in nature, because, working as a catalyst for processes, the physicochemical characteristics of the nanoparticles under study do not change, and nor does their concentration. In this respect, we made changes to the protocol for future studies, increasing the observation time from 2 days to 5 days. Any studies revealing the results of the use of nanoparticles should contain descriptions of the methods used for their preparation and the most detailed physicochemical characteristics of the actual nanochemical substances and their composites. An accurate description of the experiments performed, with the often unclear mechanisms of the effects obtained, will make it possible to repeat the published protocols and confirm or throw into question the results obtained by other researchers, which will accelerate the understanding of the place of nanomaterials in general, and nanoceria in particular, in clinical medicine.

The authors hypothesized why *E. coli* is particularly sensitive to cerium nanoparticles.

The diffusion of metal nanoparticles is very slow and does not fit within the limits of the experimental protocol, which was in accordance with the standards of the pharmacopoeia (for antibiotic and antiseptic research). This property has already been noted by researchers who even had to place nanoparticles on an agar plate under a constant electric current in order to accelerate diffusion [79]. The diffusion on agar was better if the nanoparticles had a low stability and released metal ions. Such an effect obtained from the study of silver nanoparticles was proven by X-ray diffraction analysis [80]. The same effect with ion-forming metal nanoparticles was obtained by other researchers [81]; one of the positive properties of the study of metal nanoparticles was recognized as the fact that the method allowed for the identification of nanomaterials that produce antimicrobial ions and have a synergistic effect in neutral agar medium.

The absence of growth retardation zones of Gram-positive bacteria can be explained by the structure of their cell walls, which contain multilayer peptidoglycan, protecting these microorganisms from adsorption and penetration of heavy metals into the cytoplasm. Similar effects of a lower sensitivity of Gram-positive bacteria to nanoparticles of different metals were explained by other researchers who obtained the same effect precisely by the difference in the structure of the bacterial wall [82,83].

At the same time, the results we obtained when studying the effect of nanoceria on Gram-negative bacteria (*Pseudomonas aeruginosa*, *Escherichia coli*, and *Proteus vulgaris*) are ambiguous. The same ambiguous reaction was obtained by other researchers when they studied a line of Gram-positive and Gram-negative microorganisms [84].

The use of nanoparticles of different chemical compositions could give a selective strong or weak antimicrobial effect depending on the type of pathogen [85], which can be explained by the action or inaction of nanoparticles of a particular composition on the biochemical processes occurring in the bacterial cell.

The study of how the mechanisms of antimicrobial activity of metal nanoparticles work continues to be debated, but much of these theories still lie in the realm of hypotheses [86] that will require further evidence.

We believe that the absence of a pronounced growth inhibition of *P. aeruginosa* under the action of cerium ions was due to the ability of these bacteria to form alginate mucus under unfavorable conditions, which prevents the adsorption of heavy metals on the surface of the cell wall and their penetration into the cytoplasm.

*E. coli* is not characterized by the secretion of mucus or mucus-like substances, so the negative effect of the cerium ions on these bacteria could have developed in the following way. Adsorbed on the outer membrane of Gram-negative bacteria, cerium inhibits the functional activity of protein porins, which leads to the entry of excessive concentrations of this ion into the cytoplasm. Penetrating inside the cell, the investigated metal can bind to sulfhydryl, hydroxyl, and amino groups of proteins and lipids of bacteria. Reactions of complexation with heavy metal lead to the inactivation of many enzymes, including those involved in the respiration process, and cause a number of degradative changes, resulting in both the delayed reproduction of the microorganism and its death.

*P. vulgaris* bacteria also do not form mucus under unfavorable conditions and do not differ much from *E. coli* in structure. It can be assumed that the lack of an obvious biocidal effect of cerium against Proteus was due to its biochemical properties. *P. vulgaris* has more pronounced proteolytic and peptolytic activity than *E. coli*. A typical property of *E. coli* is its ability to break down sugars. According to the classical methodology, our study was conducted using meat-peptone agar, a simple carbohydrate-free protein nutrient medium. It is likely that *P. vulgaris* secretes peptide cleavage products that bind cerium ions and prevent their entry into the cell in large quantities into the nutrient medium. *E. coli*, showing less proteolytic activity, does not form substances capable of reacting with heavy metal and inhibiting its adsorption onto the surface of the cell wall in carbohydrate-free medium.

The high sensitivity of *E. coli* specifically to nanomaterials of a metallic nature was also pointed out by other researchers [87,88,89].

The main innovation of this work was to obtain evidence that citrate-stabilized sols of cerium dioxide nanoparticles do not possess an absolute bactericidal effect; therefore, the assessment of their antimicrobial properties should not be carried out using classical methods (seeding) but through the use of precise quantitative methods, including the use of gas chromatography–mass spectrometry. For the first time, the patterns of the direct dose-dependent effect of cerium dioxide nanoparticles have been proven, which, unlike antibiotics and antiseptics, progress over time. The severity of the antimicrobial effect correlates with the redox activity, which progressively increases as the CeO_2_ concentration increases. Consequently, the antimicrobial (bacteriostatic) effects of cerium oxide nanoparticles of a new type have been proven, coupled with the peroxidase-like mechanism of action of nanoparticles with a variable valence, which can be of significant use in regenerative medicine.

## 5. Conclusions

The results of this classic study examining the antimicrobial activity of different concentrations of citrate-stabilized cerium oxide nanoparticles (methods of serial dilutions and the determination of the activity of antibiotics by diffusion in agar) did not show an absolute antimicrobial effect characteristic of pharmacopeial standards for antibiotics and antiseptics; however, indirect qualitative and quantitative signs were determined, demonstrating the antimicrobial activity of nanoceria. At the same time, the antimicrobial activity (bacteriostatic effect) against *E. coli* in all the studied nanoceria samples was determined using gas chromatography with a mass-selective detector.

A reliable dose-dependent bactericidal effect of the nanoceria was established; the higher the dose used, the more pronounced the antimicrobial activity. The most significant (15.9 times) decrease in the value of the determined indicator (the number of *Escherichia coli* ATCC 8739) was recorded in the nutrient medium with the addition of Ce^−1^ct (10^−2^); after 48 h of incubation, the nanoceria reliably suppressed the growth and number of *E. coli* after 24 h at high concentrations (by 72% at a concentration of 10^−2^ M and 28% at a concentration of 10^−3^ M at a dose of 10 vol%) and, particularly significantly, after 48 h with a wide range of concentrations (10^−2^–10^−5^ M) on average by 48–94%.

A reliable redox activity of nanoceria coated with citrate was established, increasing in proportion to the concentration, confirming the oxidative mechanism of action of nanoceria. The peroxidase-like activity of the citrate-stabilized cerium dioxide sols was most pronounced at a concentration of 10^−3^ M, which corresponded to the best bacteriostatic effect.

The antimicrobial effect was proven to increase over time, and after 48 h, the antimicrobial effect was more pronounced than after 24 h. This provides us with reason to believe that nanoceria can provide a prolonged antimicrobial effect.

The inconsistency in the data regarding the antimicrobial activity of nanoceria may be due to the limited use of standardized microbiological methods used for the development of classical antibacterial drugs, which is not true in the development and application of nanochemistry, which operates according to other laws.

When developing new nano-preparations providing new types of antimicrobial action (not standard antibiotics), it is advisable to use a wide range of microbiological methods to obtain objective and reliable data.

## Figures and Tables

**Table 1:** Zones of growth retardation (mm).

	Citrate-Coated Cerium Oxide Nanoparticles					Control	Comparison	
Microorganisms	10^−2^ M	10^−3^ M	10^−4^ M	10^−5^ M	10^−6^ M	H_2_O	Ceftriaxone	Levomekol
*B. subtilis*	0	0	0	0	0	0	62/52/64/60/59	50/33/37/50/43
*B. cereus*	0	0	0	0	0	0	27/23/28/32/25/65	50/50/48/44/50/47
*Ps. aeruginosa*	0	0	0	0	0	0	45/47/42/35/43	36/30/36/26/32
*Pr. vulgaris*	0	0	0	0	0	0	47/55/52/53/51	40/40/50/36/42
*E.coli*	16/18/2/0/25	0	0	0	0	0	60/65/63/59/57	48/45/48/50/52
*St. aureus*	0	0	0	0	0	0	40/41/38/41/46	66/46/30/40/34/33
*Candida*	0	0	0	0	0	0	40/34/32/34/30/14	45/50/45/40/40
*Aspergillus*	0	0	0	0	0	0	0	0

**Table 2:** Intensity of microorganism growth at different concentrations of citrate-stabilized cerium oxide.

Test Tube Number	1	2	3	4	5	6	7	8	9	10	11 Control
Nano-cerium concentration (g/mL)	0.05	0.025	0.0125	0.0063	0.0031	0.0016	0.0008	0.0004	0.0002	0.0001	0
*E. coli*	+++	++	++	+++	+++	+++	+++	+++	+++	+++	+++
*Candida alb.*	++	+++	+++	++	++	+++	+++	+++	+++	+++	+++
*St. aureus*	++	++	++	+++	+++	+++	+++	+++	+++	+++	+++
*B. subtilis*	+++	+	++	++	++	++	++	++	+++	+++	+++

Note: +++ maximum growth intensity; ++ average and + minimal (relative to the control and all in general) intensity of microorganism growth. The color scheme in the table cells corresponds to the color change in the test tubes of the dilution method.

**Table 3:** Descriptive statistics of gas chromatography results for *E. coli* growth inhibition at 24 and 48 h.

	% Suppression Relative to Control	Mean	Std. Deviation	Std. Error	95% Confidence Interval for Mean		Minimum	Maximum
					Lower Bound	Upper Bound		
24 h								
CM + *E. coli*	Control	341.0	6.7	2.8	333.9	348.1	332	347
CM + *E. coli* + H_2_O	Control	341.0	8.3	3.4	332.3	349.7	329	348
Ceftriaxone	100%	0.0	0.0	0.0	0.0	0.0	0	0
CeOct (10^−2^ M)	72%	96.5	2.6	1.1	93.8	99.2	94	100
CeOct (10^−3^ M)	28%	244.3	4.4	1.8	239.7	249.0	239	251
CeOct (10^−4^ M)	-	335.2	5.7	2.3	329.1	341.2	327	342
CeOct (10^−5^ M)	-	336.5	9.0	3.7	327.0	345.9	322	350
48 h								
CM + *E. coli*	Control	2659.20	49.55	22.15	2597.68	2720.72	2622	2743
CM + *E. coli* + H_2_O	Control	2612.83	223.45	91.22	2378.34	2847.33	2164	2759
Ceftriaxone	100%	0.0	0.0	0.0	0.0	0.0	0	0
CeOct (10^−2^ M)	94%	170.40	5.51	2.46	163.57	177.23	165	178
CeOct (10^−3^ M)	76%	634.20	3.89	1.74	629.36	639.04	630	640
CeOct (10^−4^ M)	59%	1098.80	11.56	5.17	1084.44	1113.16	1086	1111
CeOct (10^−5^ M)	48%	1381.40	16.39	7.33	1361.04	1401.76	1361	1401

## Data Availability

Data are contained within the article.
